# Supplementary material for: Remedial colon hydrotherapy device enema as a salvage strategy for inadequate bowel preparation for colonoscopy: A retrospective cohort study
Source: PLoS One. 2025 Mar 19;20(3):e0319493. doi: 10.1371/journal.pone.0319493 (PMC11922272; doi:10.1371/journal.pone.0319493)
Supplement: S2 File — (DOCX) [file pone.0319493.s002.docx]

****Telephone Follow-up Survey****

****Introduction:****

"Hello, my name is [Dongxuan Zhang], and I'm calling from Beijing Changping Hospital of Traditional Chinese Medicine. We are currently conducting a brief survey on the optimal remedial strategy for patients with inadequate bowel preparation prior to colonoscopy. We reviewed your medical records and found that you had a colonoscopy at our endoscopy centre in [Time]. You also had a remedial bowel preparation following a routine bowel preparation prior to the colonoscopy.Your participation is very important to us. Would you be able to spare a few minutes to answer some questions?"

****Participant Information:****

"Could you please confirm your identity for our records, is your name [Name]?"

"May I start by asking for your age and gender, please?"

Name

Age

Gender

****Main Questions:****

1. Do you still clearly recall the process of your colonoscopy examination conducted at our endoscopy center in [Time] ?

Yes

No

1. Given that you underwent remedial bowel preparation for a previous colonoscopy due to inadequate preparation, would you prefer the same remedial preparation method for any future colonoscopies where bowel preparation is suboptimal?

Select again

Rejected

Unable to select

1. On a scale from 0 to 10, where 0 is very dissatisfied and 10 is very satisfied, how would you rate the time consumed in the remedial bowel preparation process?

| 0 | 1 | 2 | 3 | 4 | 5 | 6 | 7 | 8 | 9 | 10 |
| --- | --- | --- | --- | --- | --- | --- | --- | --- | --- | --- |
|  |  |  |  |  |  |  |  |  |  |  |

1. On a scale from 0 to 10, where 0 is very dissatisfied and 10 is very satisfied, how satisfied were you with the level of discomfort you experienced from the various symptoms during the remedial bowel preparation process?

| 0 | 1 | 2 | 3 | 4 | 5 | 6 | 7 | 8 | 9 | 10 |
| --- | --- | --- | --- | --- | --- | --- | --- | --- | --- | --- |
|  |  |  |  |  |  |  |  |  |  |  |

1. On a scale from 0 to 10, where 0 is very dissatisfied and 10 is very satisfied, how satisfied were youwith the service attitude of the medical staff during the remedial bowel preparation process？

| 0 | 1 | 2 | 3 | 4 | 5 | 6 | 7 | 8 | 9 | 10 |
| --- | --- | --- | --- | --- | --- | --- | --- | --- | --- | --- |
|  |  |  |  |  |  |  |  |  |  |  |

1. Could you please detail the discomfort you encountered during your remedial bowel preparation ?
2. Have you encountered any issues or difficulties that you would like to share?

We sincerely appreciate your invaluable contribution. The information you've shared will significantly enhance our research, and we are committed to using these findings to improve healthcare services. Rest assured, we will adhere to the highest standards of data protection and privacy throughout our study. Thank you again for your participation – it means so much to us!
