# Supplementary material for: Remedial colon hydrotherapy device enema as a salvage strategy for inadequate bowel preparation for colonoscopy: A retrospective cohort study
Source: PLoS One. 2025 Mar 19;20(3):e0319493. doi: 10.1371/journal.pone.0319493 (PMC11922272; doi:10.1371/journal.pone.0319493)
Supplement: S3 File — (DOCX) [file pone.0319493.s003.docx]

The Colon Hydrotherapy Instrument, model DJS-C, is manufactured by Hangzhou Hercules Medical Instrument Co., Ltd., in China.

The procedure was conducted in the enema room by a nurse who had completed the requisite training. Prior to commencing the procedure, the nurse informed the patient of the general procedure of the colon hydrotherapy machine, obtained the patient's understanding and co-operation, and instructed the patient to lie in the left lateral position.

(1) Initiate the power supply;

(2) Activate the ready button to set the water temperature and working flow rate (36-36.5°C, 0.7-0.8);

(3) Connect the pipeline, insert the rectal catheter into the subject's anus, and verify the security of the pipeline connection.

(4) Terminate the ready state by pressing the water inlet button. This initiates the flow of warm water into the rectal conduit from the inlet pipe, subsequently entering the drainage system via the rectal conduit, outlet pipe, and observation pipe.

(5) Activate the flushing and draining button to initiate the flushing and irrigation process. Monitor the pipeline pressure throughout this process.

In the process of flushing and discharging, the operator can ascertain that the amount of water in the subject's colon is sufficient based on the following observations:

a. Subject's reaction: the subject indicates that the colon is filled, at which point the operator should terminate the filling and discharging state, and proceed to discharge the contents.

Once the colon has been sufficiently filled, the operator must enter the discharging state, eliminate the accumulated faecal matter, and then re-enter the filling and discharging state. This process must be repeated until the accumulated faecal matter has been eliminated.

b. The pressure is displayed: the system activates the safety protection function and ceases the water intake when the pressure is greater than 10 KPa.

(6) Steps 4 and 5 should be repeated until the patient's colon is irrigated with a watery stool or a yellowish transparent liquid.

(7) Once the enema is complete, the rectal catheter, inlet pipe and outlet pipe should be dismantled, the equipment cleaned and disinfected.
