# Supplementary material for: Remedial colon hydrotherapy device enema as a salvage strategy for inadequate bowel preparation for colonoscopy: A retrospective cohort study
Source: PLoS One. 2025 Mar 19;20(3):e0319493. doi: 10.1371/journal.pone.0319493 (PMC11922272; doi:10.1371/journal.pone.0319493)
Supplement: S4 File — (DOCX) [file pone.0319493.s004.docx]

# The Boston Bowel Preparation Scale

The BBPS is a standardized 9-point assessment scale for the colon. The structure of the colon is divided into its three segments: right colon, transverse colon, and left colon. Each segment is classified from 0 to 3 depending on the degree of soiling. The sum total of the three segments represents the degree of soiling, so that a total ≤ 5 points shows poor bowel preparation, while 6–7 shows good bowel preparation, and ≥ 8 very good bowel preparation.

| **Grade** | **Grade Endoscopic findings** |
| --- | --- |
| 0 | Represents an unprepared colon segment. The mucosa is not visible due to solid feces, and the fecal masses cannot be removed |
| 1 | Parts of the mucosa in the colon segment can be seen, but other areas of the same segment cannot be seen well due to blockage with stool and/or opaque liquid. |
| 2 | The mucosa of the colon segment can be seen well, with minor amounts of residual stool, small fragments of stool and/or opaque liquid. |
| 3 | The entire mucosa of the colon segment can be seen well, with no residual soiling. |
