# Supplementary material for: Remedial colon hydrotherapy device enema as a salvage strategy for inadequate bowel preparation for colonoscopy: A retrospective cohort study
Source: PLoS One. 2025 Mar 19;20(3):e0319493. doi: 10.1371/journal.pone.0319493 (PMC11922272; doi:10.1371/journal.pone.0319493)
Supplement: Attachment — Submitted filename: A rebuttal letter.docx [file pone.0319493.s007.docx]

Dear Editors and Reviewers:

We are writing to express our sincere appreciation for your insightful comments and suggestions regarding our article entitled “Remedial colon hydrotherapy device enema as a salvage strategy for inadequate bowel preparation for colonoscopy: A retrospective cohort study” (Manuscript No: PONE-D-24-32926). This study explores remedial strategies to improve bowel preparation for colonoscopy, a critical aspect of effective diagnostic procedures. The constructive feedback provided by the reviewers has been invaluable in enhancing the quality of our manuscript. We have made extensive modifications and supplemented additional data to strengthen our findings and ensure the robustness of our results. All changes in the revised manuscript are highlighted in red text for your convenience. Below this letter, you will find point-by-point responses to the comments from the three reviewers. We believe these revisions have significantly improved our manuscript, and we are grateful for the opportunity to address these important issues.

**Reviewer #1**

**Major concerns:**
**1. Line 111: The extended time between the colonoscopy exam and the follow-up phone call may affect the patient's ability to recall details of the preparation and procedure. What were the phone call questionnaires?**

**The author's answer**: Thank you for your valuable comments. We apologize for not uploading the telephone questionnaire as an attachment when the manuscript was first submitted. The telephone questionnaire in this study consisted of three parts.

The first part was the Introduction, which explained the purpose and content of the telephone follow-up to the respondents and obtained their consent to proceed with the questionnaire. The second part aimed to confirm the respondent's basic personal information, including name, age, and gender. The third part consisted of seven main questions designed to gather detailed insights relevant to our study. These questions were as follows:

① Do you still clearly recall the process of your colonoscopy examination conducted at our endoscopy center in [Time]?

② Given that you underwent remedial bowel preparation for a previous colonoscopy due to inadequate preparation, would you prefer the same remedial preparation method for any future colonoscopies where bowel preparation is suboptimal? (Select again, Rejected, Unable to select)

③ On a scale from 0 to 10, where 0 is very dissatisfied and 10 is very satisfied, how would you rate the time consumed in the remedial bowel preparation process?

④ On a scale from 0 to 10, where 0 is very dissatisfied and 10 is very satisfied, how satisfied were you with the level of discomfort you experienced from the various symptoms during the remedial bowel preparation process?

⑤ On a scale from 0 to 10, where 0 is very dissatisfied and 10 is very satisfied, how satisfied were you with the service attitude of the medical staff during the remedial bowel preparation process?

⑥ Could you please detail the discomfort you encountered during your remedial bowel preparation?

⑦ Have you encountered any issues or difficulties that you would like to share?

These questions were designed to assess the respondents' experiences and satisfaction with the remedial bowel preparation process. We uploaded the telephone questionnaire as supplementary data, as S2 File. Meanwhile we have a statement in the manuscript at lines 218 to 225.

In conducting this retrospective study, we included patients who underwent remedial bowel preparation between April 2019 and June 2022 to ensure an adequate sample size for inclusion. The telephone questionnaire was conducted between October 2022 and December 2023, which introduced the potential for recall bias in the results.To minimize recall bias, our team members reviewed the patients' inpatient medical records to identify any discrepancies with the telephone questionnaire responses. This cross-verification helped ensure the accuracy of the data collected. To further address recall bias, we are planning a prospective study. This study will aim to collect information from patients immediately after they have received bowel preparation, thereby reducing the reliance on long-term memory and improving data accuracy.

2. **Line 123 -130: The BBPS score or stool assessment prior to salvage cleansing should be presented.**

**The author's answer**: We appreciate your valuable comments. In this retrospective study, the degree of bowel cleansing prior to remedial bowel preparation was assessed based on the fecal characteristics of the last bowel movement before the proposed colonoscopy. The assessment was graded into four categories:

Grade 1: Very poor bowel preparation (brown fecal water with a large amount of waste material).

Grade 2: Relatively poor (dark yellow fecal water with a small amount of fecal waste material).

Grade 3: Good (yellowish clear watery stool).

Grade 4: Very good (colorless clear watery stool).

Grades 1 and 2 were considered inadequate bowel preparation. We have revised and clarified lines 125-135 of the article to better explain this grading system. The results of the stool assessment prior to bowel preparation for each patient are provided in the S1 file.

In the manuscript, we conducted a statistical analysis of the pre-remedial bowel preparation fecal assessment results for both groups. The overall distribution was similar, with no statistical difference observed (χ² = 0.451, *P* = 0.502). This analysis is presented in lines 248-251 of the manuscript, and the statistical results are detailed in Table 1.

3. **Line 128-130: Is the BBPS score assessed during the first colonoscopy view, and if BBPS score <6 or <2 in any segment, is salvage cleaning then applied? Please clarify this point.**

**The author's answer**: We thank you for your helpful comments and apologize for not explaining this issue clearly in the article. In our daily clinical practice at the Gastroenterology Department and Endoscopy Centre, we perform an initial assessment of bowel cleanliness for patients undergoing colonoscopy on the day of the scheduled examination. This assessment is based on the patient's last bowel movement, and the results are recorded in the patient's medical record. For patients assessed as Grade 1 or Grade 2, we inform them of the potential adverse effects of poor bowel preparation and recommend remedial bowel preparation, while ultimately respecting the patient's choice.

In this retrospective study, we did not use the Boston Bowel Preparation Scale (BBPS) score to decide whether a patient should undergo remedial bowel preparation. During data collection, we found that only 3 of 121 patients with a left hemicolon BBPS score of less than 2 at the start of the colonoscopy were withdrawn and underwent remedial bowel preparation at the end of the procedure. The final 109 patients included in the study were judged to have failed bowel preparation based solely on their final bowel movement after routine preparation. We apologize for not detailing this in the article and are correcting and clarifying this information on lines 125-135 and lines 155-159.

4. **Line 142: The timing and method for assessing bowel cleanliness before the salvage cleaning preparation were not provided in the article.**

**The author's answer:** Thank you for pointing out this issue in the manuscript. We apologize for not explicitly stating the timing and method for assessing bowel cleanliness prior to the salvage cleaning preparation in the manuscript. We have now detailed and revised this information in lines 125-135. In our daily clinical practice at the Gastroenterology Department and Endoscopy Centre, we perform an initial assessment of bowel cleanliness for patients undergoing colonoscopy on the day of the scheduled examination. This assessment is based on the patient's last bowel movement, and the results are recorded in the patient's medical record. For patients assessed as Grade 1 or Grade 2, we inform them of the potential adverse effects of poor bowel preparation and recommend remedial bowel preparation, while ultimately respecting the patient's choice.

5. **Line 184, 363-364: What are the questionnaires consisted in the phone calls? Please provide the questionnaires and chart as supplement data. How is the patient satisfaction score graded? Is the Leaker Score used?**

**The author's answer:** We appreciate your pointing out these issues in the manuscript, and we sincerely apologize for not addressing the issues you raised in the manuscript. We uploaded the telephone questionnaire as supplementary data, as S2 File. The telephone questionnaire consisted of seven questions. The tool used for the satisfaction assessment part of the telephone follow-up was the Likert scale, which assessed patients' satisfaction with the bowel preparation process by rating the following three areas: (a) satisfaction with the time taken for the bowel preparation process; (b) tolerance of discomfort during the bowel preparation process; and (c) satisfaction with the service attitude of the medical staff during the bowel preparation process. Satisfaction was rated on a numerical scale from 0 (very dissatisfied) to 10 (very satisfied).

6. **Line 213: The difference between BBPS scores of 7 and 6.5 is minimal, especially given the study's small sample size. Hydrotherapy appears to be most effective in cleaning the descending colon, as shown in Table 2, but it seems to have limited impact on the ascending and transverse colons. Therefore, it is recommended to increase the sample size to enhance the reliability of the observed differences.**

**The author's answer:** Thank you for your valuable comments. We fully understand your concerns. The sample size of this retrospective study is indeed small, and there are unavoidable issues such as recall bias and incomplete data. To improve the reliability of the observed differences and to reduce these common issues in retrospective studies, our team is continuing to conduct relevant prospective studies. We aim to collect more subjects with complete data to support our research findings.

7. **Line 229: How is the success rate defined? Was cleanliness graded and compared before and after salvage cleaning? What is the average experience in years of the colonoscopists? Please provide the actual data in chart or supplementary data.**

**The author's answer:** Thank you for your question. We sincerely apologize for not defining "success rate" within the manuscript. In this study, the Boston Bowel Preparation Scale (BBPS) was utilized to evaluate the quality of bowel preparation in patients undergoing salvage bowel preparation. The BBPS is a standardized 9-point scale for assessing the colon, which is divided into three segments: right colon, transverse colon, and left colon. Each segment is rated from 0 to 3 based on the degree of cleanliness. The total score, which ranges from 0 to 9, indicates the level of bowel preparation: ≤5 points indicates poor preparation, 6–7 points indicates good preparation, and ≥8 points indicates very good preparation. A BBPS score of ≥6 was considered satisfactory. The success rate was defined as the proportion of patients in each group achieving a BBPS score of ≥6. Additionally, differences in BBPS scores across bowel segments and overall scores were compared between the two groups. The definition of success rate has been added to the manuscript at lines 204-205. To facilitate understanding of the scale content, we have uploaded the BBPS as supplementary data, designated as S4 File.

In this retrospective study, BBPS scores for patients were not recorded before remedial bowel preparation. the degree of bowel cleansing prior to remedial bowel preparation was assessed based on the fecal characteristics of the last bowel movement before the proposed colonoscopy. The assessment was graded into four categories:

Grade 1: Very poor bowel preparation (brown fecal water with a large amount of waste material).

Grade 2: Relatively poor (dark yellow fecal water with a small amount of fecal waste material).

Grade 3: Good (yellowish clear watery stool).

Grade 4: Very good (colorless clear watery stool).

Grades 1 and 2 were considered indicative of inadequate bowel preparation. For patients assessed with these grades, we inform them of the potential adverse effects of insufficient bowel preparation and recommend remedial measures, while ultimately respecting the patient's decision. The results of the stool assessment prior to bowel preparation for each patient are provided in the S1 file. In the manuscript, we conducted a statistical analysis of the pre-remedial bowel preparation fecal assessment results for both groups. The overall distribution was similar, with no statistical difference observed (χ² = 0.451, *P* = 0.502). This analysis is presented in lines 249-251 of the manuscript, and the statistical results are detailed in Table 1.

Our endoscopy center currently employs five endoscopists. One has 20 years of experience, while the remaining four have 7, 8, 10, and 4 years of experience, respectively.

8. **It would be interesting to compare the ADR between the two groups.**

**The author's answer:** Thank you for your valuable comments. We have compared the ADR between the two groups in the results section (line 291-303). The statistics are presented in Table 5, along with Fig 2, which provides a more visual comparison of the ADR between the two groups.

**Fig 2. Adverse reaction incidence in remedial bowel preparation between two patient groups** ns, no significance; *, P ≤ 0.05; **, P ≤ 0.01; ***, P ≤ 0.001

9. **Line 358-360: The supplement data or chart did not provide information on conditions such as carotid atherosclerosis, hypertension, cerebral infarction, and numerous other cardiovascular and cerebrovascular diseases. On what basis was this conclusion made?**

**The author's answer:** Thank you for your valuable comments. In this retrospective study, we observed that 5 patients in the hydropathy enema group and 3 in the oral medication group experienced dizziness. Such reactions could lead to dangerous situations like falls, prompting further analysis of these patients' case records to identify common characteristics. We found that the average age of these 8 patients was 74.25±5.6 years, and all had a history of cardiovascular and cerebrovascular diseases. Therefore, we reported in the article, "The majority of patients who experienced dizziness during remedial bowel preparation were of advanced age and presented with a combination of carotid atherosclerosis, hypertension, cerebral infarction, and several other cardiovascular and cerebrovascular diseases." The clinical diagnoses of all patients are detailed in S1 file, and we compiled the clinical information of these 8 patients into S5 file. We will continue prospective studies to clarify the incidence and risk factors associated with dizziness as an adverse reaction during remedial bowel preparation.

10. **The BBPS score shown in the S1 file assessed after applying salvage strategy?**

**The author's answer:** Thank you for your question. Yes. After remedial bowel preparation, the patient's bowel cleanliness was assessed using the Boston Bowel Preparation Scale (BBPS).

**Minor concerns:**
**1. English writing skills should be improved.**

**The author's answer:** Firstly, I would like to express my sincere gratitude for the feedback you have provided. We value your comments on improving our English writing skills. We recognize that there are deficiencies in the English expression in this submission, which may have impacted the clarity and professionalism of the paper. We sincerely apologize for this and promise to take steps to improve. To enhance the quality of the paper, we have engaged a professional English editor to conduct a thorough language review of the entire text. Additionally, we plan to use advanced language correction software to aid future writing efforts, ensuring that submitted papers meet the highest linguistic standards. And here we did not list the changes but marked in red in the [revised paper](https://zhida.zhihu.com/search?content_id=238622203&content_type=Article&match_order=1&q=revised+paper&zhida_source=entity). We are committed to continuously improving our English writing skills in future research and writing, ensuring that our work meets the standards of the journal.

Thank you again for your feedback and support.

2. **The make and model of the hydrotherapy machine should be included in the method or supplementary S2 file.**

**The author's answer**: Thanks for your suggestion. The Colon Hydrotherapy Instrument, model DJS-C, is manufactured by Hangzhou Hercules Medical Instrument Co., Ltd., in China. We have added this information to the Methods section (lines 143-144) and the S3 file.

3. **The labels in Table 2 are unclear. “Hepatic, transverse and splenic flexure” is confusing.**

**The author's answer**:Thank you for your valuable comments. We recognize that our presentation in the article can indeed be confusing. The Boston Bowel Preparation Scale (BBPS) is a four-point scoring system applied to each of the three broad regions of the colon: the right colon (including the cecum and ascending colon), the transverse colon (including the hepatic and splenic flexures), and the left colon (including the descending colon, sigmoid colon, and rectum). To enhance clarity and understanding, we have made explanations and changes in the manuscript. An explanation of the BBPS has been added to lines 196-204. In the manuscript, "the descending, sigmoid, and rectal segments" has been rewritten as "the left colon" and "the hepatic and splenic flexures" has been rewritten as "the transverse colon" Similarly, "the cecum and ascending colon" has been changed to "the right colon". The labels in Table 2 have been updated accordingly.

4. **Line 342-343: redundant, repeated and unclear statement.**

**The author's answer:** We would like to express our gratitude for your insightful comments, which have informed the revisions we have made to the manuscript. We have deleted repetitive statements (lines 399-400).

5. **Reduce the use of long sentences. For example in line 394 to 399.**

**The author's answer:** We think this is an excellent suggestion. We have revised the entire manuscript to reduce the use of long sentences, aiming for clarity and brevity of expression. The revised statements are labelled in the article.

6. **In S1 file, cases number 25, 71, and 82: The “colonoscopy” column indicates “poor bowel preparation”, yet the BBPS score is still 6. Please clarify.**

**The author's answer:** Thank you for your comments. We reviewed the colonoscopy images and BBPS scores of these three patients and confirmed that their scores were 6. However, there was a statement of poor bowel preparation in the endoscopy report. After communicating with the endoscopist who authored the report, the following explanation was provided: Colonic polyps were found in patient 25 and patient 71, and endoscopic treatment was recommended. Remedial bowel preparation was carried out after their initial poor preparation. The statement about inadequate bowel preparation was included to encourage patients to improve their bowel preparation and compliance before their next endoscopy. Notably, patient 82 was an elderly patient with chronic constipation following colon cancer surgery, who regularly underwent colonoscopy reviews at our center. Although these patients achieved a BBPS score of 6 after remedial preparation, the report noted poor preparation to prompt regular reviews and emphasize the importance of improving bowel preparation cleanliness.
**7. Please review all the punctuation.**

Thank you for your careful reading and valuable comments. We have checked the punctuation of the manuscript and revised it accordingly. The revisions are indicated in the manuscript.

**Reviewer #2**

**Comments about the preparation:
· There is a detailed explanation about second preparations, but not about the main protocol of colon preparation. All patients followed a standard protocol? Could there be differences?**

**The author's answer:** We appreciate your insightful comments and apologize for the insufficient detail in the initial bowel preparation protocol presented in the manuscript. The standard bowel preparation protocol at our endoscopy center prior to colonoscopy is the 3 L PEG protocol, which is categorized into a single-dose PEG protocol and a split-dose PEG protocol. Considering both national and international literature and the characteristics of our population, the risk factors for inadequate bowel preparation in China include chronic constipation, a BMI greater than 25 kg/m², age over 70 years, a history of colon surgery, and the presence of comorbidities such as diabetes mellitus, Parkinson's disease, stroke, or spinal cord injury. Additionally, the use of tricyclic antidepressants or anesthesia also poses a risk. For patients with these risk factors, we recommend administering 1 L of the preparation 6 to 10 hours before the examination and 2 L 4 to 6 hours before the examination on the day of the procedure. For patients lacking clear risk factors, we advise consuming 3 L of the preparation 4 to 6 hours before the examination on the day of the procedure. We have included and described the initial bowel preparation protocol in the manuscript (lines 123-135). Additionally, we have supplemented the S1 file with detailed information on the initial bowel preparation protocol for each patient. We also conducted a statistical analysis of the data on initial bowel preparation protocols. In the hydrotherapy machine enema group, 47 patients followed the 3 L PEG split oral protocol, and 8 patients followed the 3 L PEG single protocol. In the oral medication group, 43 patients utilized the 3 L PEG split oral protocol, while 11 patients used the 3 L PEG single protocol. The proportions of the two initial bowel preparation protocols in both groups were comparable, with no significant statistical difference (χ² = 0.642, *P* = 0.423). We have referenced this analysis in line 248-251 of the manuscript, where the statistics and comparisons of bowel preparation protocols between the two patient groups are detailed, and the corresponding data is presented in Table 1.

**· Was there any evaluation of previous experience in colonoscopy?**

**The author's answer:** We appreciate your valuable comments on the manuscript. Yes. In our daily clinical practice at the Gastroenterology Department and Endoscopy Centre, we perform an initial assessment of bowel cleanliness for patients undergoing colonoscopy on the day of the scheduled examination. This assessment is based on the patient's last bowel movement, and the results are recorded in the patient's medical record. The assessment was graded into four categories:

Grade 1: Very poor bowel preparation (brown fecal water with a large amount of waste material).

Grade 2: Relatively poor (dark yellow fecal water with a small amount of fecal waste material).

Grade 3: Good (yellowish clear watery stool).

Grade 4: Very good (colorless clear watery stool).

Grades 1 and 2 were considered inadequate bowel preparation. For patients assessed with Grades 1 and 2, we inform them of the potential adverse effects of insufficient bowel preparation and recommend remedial measures, while ultimately respecting the patient's decision. The results of the stool assessment prior to bowel preparation for each patient are provided in the S1 file. We have revised and clarified lines 125-135 of the manuscript to better explain this grading system.

**· If I understand correctly, patients with inadequate colon preparation have to do a second treatment after a colonoscopy. So after sedation, they are informed and make a choice between hydrotherapy and oral medication. How much time passes between they awake and start the second preparation?**

**The author's answer:** Thank you for your valuable comments. We apologize for any lack of clarity in the manuscript. Painless gastrointestinal endoscopy was only introduced at our endoscopy center in 2023, and in this retrospective study, patients underwent colonoscopy without anesthesia. After stool assessment, patients judged to have inadequate bowel preparation were given additional preparation with their consent, as advised by the attending physician. Since this was a retrospective study, our assessment of timing relied primarily on patients' medical records and time information from the endoscopy information system. During data collection and analysis, we extracted patients' medical records and the start time of post-remedial colonoscopy to evaluate the timing of remedial bowel preparation. This explanation is provided in lines 206-213 of the manuscript. Acknowledging the limitations in accuracy inherent in retrospective studies, our team has proceeded with prospective clinical trials to further validate our findings.

**Comments about the patients, selection criteria and potential bias:
· How many patients were excluded of the final evaluation? Criteria is explained, but there is no further information.**

**The author's answer:** We appreciate your valuable comments and apologize for not addressing this issue in the manuscript. During our retrospective study, a total of 121 patients were enrolled for bowel preparation remediation due to inadequate bowel preparation. Of these, 3 patients did not complete colonoscopy due to difficulties with scope access or intolerable abdominal pain, and 9 patients refused to participate in telephone follow-up. Consequently, 109 patients were included in the final study, as detailed in lines 136-146 of the manuscript. We have also provided a flowchart (Figure 1) to illustrate the process.

**Fig 1. The flow chart**

**· It is a very important limitation the N of patients and do not allow subgroups. As we see in the S1 file, their baseline characteristics in medical antecedents are very different. For example, there are patients that have incomplete intestinal obstruction, but no information of the level is given. Further, history of abdominal surgery is a very vague term. As we can see in the file, there are included in a same group colorectal tumor surgeries, gynecological surgeries, etc. without differentiation. They are important details for colonic transit and preparation results.**

**The author's answer:** Thank you for your valuable comments. Our regression study ultimately included 109 individuals, which represents a small sample size and can influence the analysis of the results. We understand and share your concern regarding this issue. Therefore, based on the preliminary results of this retrospective study, our team is currently conducting prospective clinical trials to increase the sample size, reduce potential biases inherent in retrospective studies, and validate our conclusions. We assessed the baseline characteristics of both groups concerning sex, age, BMI, ALD, underlying conditions, history of pelvic-abdominal surgery, and stool assessment, found no statistically significant differences. However, some of these indicators were initially defined with insufficient detail and were somewhat vague. Based on your valuable feedback, we have further enhanced and clarified the baseline characteristics of the two patient groups. Some patients were discharged with a diagnosis of incomplete bowel obstruction. We reviewed their medical records and found that the diagnostic criteria included the presence of abdominal pain, symptoms of abdominal distention, decreased bowel movements and stool evacuation, along with abdominal examinations and imaging indicating reduced pneumoperitoneum in the bowel lumen. Improvement of symptoms after hospitalization with conservative treatment was also part of the criteria. We acknowledge that, within the current medical environment, our diagnostic criteria for incomplete bowel obstruction are fairly broad and imprecise. Diagnosis largely relies on the patient's clinical manifestations combined with abdominal imaging studies. A total of 29 patients were diagnosed with incomplete bowel obstruction, with 26 of them also diagnosed with constipation upon reanalysis of their medical records. We also found that a greater number of patients diagnosed with incomplete bowel obstruction opted for hydrotherapy device enemas as a remedial bowel preparation method, likely due to a combination of factors involving the patient, the supervising physician, and other considerations. Your valuable comments, highlighting potential biases inherent in retrospective studies, have reinforced our awareness of the necessity for prospective studies to obtain more accurate and comprehensive insights. In response to your valuable comments, we refined the comparison of patients' baseline characteristics by separately analyzing the history of different abdominal surgeries, including colorectal, gallbladder, uterus and adnexa, and appendix surgeries, between the two groups. A chi-square test was applied, revealing comparable proportions of patients with histories of these surgeries between the groups, with no statistically significant differences. The statistical results are detailed in the manuscript (lines 246-251), and Table 1 presents the data from the analysis.

**· No information about baseline medications is given, and they may influence on final results of preparations.**

**The author's answer:** We sincerely appreciate the valuable comments. Following your suggestion, we conducted a retrospective analysis of medication records for all included patients. Our literature review identified the most common medications that may influence bowel preparation: calcium antagonists, non-steroidal anti-inflammatory drugs, anxiolytics, diuretics, statins, and hypoglycemic agents such as metformin and glycosidase inhibitors. We performed a statistical analysis using the chi-square test to compare the prevalence of these medications between the two groups. Our results indicated no significant differences in the proportions of patients taking oral calcium antagonists, non-steroidal anti-inflammatory drugs, anxiolytics, diuretics, statins, metformin, or glycosidase inhibitors between the groups. These findings are described in the manuscript (lines 80-86, 247-251), with detailed statistical results presented in Table 1.

**Medication history of patients in both groups**

| Variables | Total (n = 109) | Hydrotherapy device enema group (n = 55) | Oral medication group (n = 54) | Statistic | *P* |
| --- | --- | --- | --- | --- | --- |
| Calcium antagonists | 34(31.1) | 18(32.7) | 16(29.6) | χ²=0.122 | 0.837 |
| NSAIDs | 22(20.2) | 8(14.5) | 14(25.9) | χ²=2.191 | 0.159 |
| Anxiolytic and antidepressant drugs | 10(9.2) | 6(10.9) | 4(7.4) | χ²=0.401 | 0.742 |
| Diuretics | 20(18.3) | 7(12.7) | 13(24.1) | χ²=2.342 | 0.144 |
| Statins | 24(22.0) | 11(20.0) | 13(24.1) | χ²=0.263 | 0.650 |
| Metformin | 17(15.6) | 8(14.5) | 9(16.7) | χ²=0.093 | 0.797 |
| Glycosidase inhibitors | 20(18.3) | 12(21.8) | 8(14.8) | χ²=0.892 | 0.459 |

**· Hydrotherapy or oral medication were personal choices, although there are some that said they were unable to make a choice. What was the reason in these cases? Was there any evaluation of preparation failure in previous studies? This may influence on personal preferences and experience of the patient.**

**The author's answer:** Thank you for your inquiries. To begin, I will address your initial question. During the telephone follow-up, we aimed to accurately gauge patients' satisfaction with their bowel preparation. We employed a numerical satisfaction scale to have patients rate their experience based on time spent, tolerance of discomfort, satisfaction with healthcare staff, and willingness to use the same procedure for future colonoscopies. Some patients were hesitant to express whether they would choose the same bowel preparation method again or indicated an inability to decide, though they did not explicitly state the reasons for their uncertainty. Your inquiry into the reasons behind this hesitation is indeed a valuable and intriguing avenue for further analysis. We conducted a further analysis of the results from the patients' telephone follow-up and their basic information. Grouping patients based on their decisions revealed no significant differences in age, gender, or bowel cleanliness pass rate after remedial bowel preparation across the three groups. However, there were differences in the incidence of adverse reactions, the Boston Bowel Preparation Scale (BBPS) score after remedial preparation, the time consumption satisfaction score, the tolerance of discomfort symptoms score, and the satisfaction with healthcare staff score. Patients in the "inability to choose" group had an overall adverse reaction incidence rate of 89%, significantly higher than patients in the "re-choice" group. Furthermore, scores for time consumption satisfaction, tolerance of discomfort, and satisfaction with healthcare staff were higher for the "re-choice" group compared to both the "refusal" and "inability to choose" groups. Based on these findings, our team hypothesizes that the high rate of adverse reactions and lower satisfaction with bowel preparation in the "inability to choose" group may contribute to their indecision, possibly influenced by individual personality traits. These findings are described in the manuscript (lines 410-421), with detailed statistical results presented in Table 8.

Table8.Comparison of characteristics between patients with three different options

| Variables | Select again (n = 57) | Rejected (n = 24) | Unable to select (n = 28) | Statistic | *P* |
| --- | --- | --- | --- | --- | --- |
| Age, Mean ± SD^a^ | 66.30±10.41 | 64.54±9.80 | 67.79±9.07 | F=0.686 | 0.506 |
| Sex, (m/f) | 30/27 | 12/12 | 17/11 | χ²=0.705 | 0.739 |
| BBPS | 7(2) | 6(3)* | 6.5(2) | Z=8.747 | 0.013 |
| The success rates of remedial bowel preparation | 87.8% | 70.8% | 85.7% | χ²=3.403 | 0.217 |
| Overall Adverse Reactions | 35.1% | 95.8% | 89.3% | χ²=38.177 | ＜ 0.001 |
| Time Consumption Satisfaction M (IQR) | 9 (1.5) | 6(3)* | 8(1)* | Z=38.78 | ＜ 0.001 |
| Tolerance of discomfort symptomsM (IQR) | 10 (1) | 5(1)* | 7 (1)* | Z=75.436 | ＜ 0.001 |
| Satisfaction with healthcare staff M (IQR) | 10 (2) | 7 (4)* | 10 (2)* | Z=41.915 | ＜ 0.001 |

* Statistically different compared to the Select again group

Our response to your second question aligns with yours. Several prospective studies and systematic reviews have evaluated bowel preparation failure. Notable studies include "A Predictive Model Identifies Patients Most Likely to Have Inadequate Bowel Preparation for Colonoscopy" (Clin Gastroenterol Hepatol), "Association Between Bowel Habits and Quality of Bowel Preparation for Colonoscopy" (Medicine [Baltimore]), and "Constipation, Fiber Intake, and Non-Compliance Contribute to Inadequate Colonoscopy Bowel Preparation: A Prospective Cohort Study" (J Dig Dis). Additionally, studies such as "Predictors of Suboptimal Bowel Preparation Using 3L of Polyethylene Glycol for an Outpatient Colonoscopy: A Prospective Observational Study" (Dig Dis Sci), "Risk Factors for Inadequate Bowel Preparation: A Validated Predictive Score" (Endoscopy), and "Patient Characteristics Associated with Quality of Colonoscopy Preparation: A Systematic Review and Meta-Analysis" (Clin Gastroenterol Hepatol) further contribute to understanding factors affecting bowel preparation quality. These studies primarily examined and summarized the risk factors for bowel preparation failure and developed predictive models. Polyethylene glycol electrolyte solution is the most commonly used bowel cleanser. The initial bowel preparation protocol typically involves either a single or divided oral dose of polyethylene glycol electrolyte solution. Unsatisfactory bowel preparation outcomes can significantly influence a patient's decision to choose the same preparation method again.

**· Follow-up interviews were done after one year or more. Recall bias is a huge limitation here.**

**The author's answer:** Thank you for your valuable comments. We share your concern that recall bias is a significant limitation of our retrospective study. To address this issue, our team is currently developing a prospective randomized controlled trial on bowel preparation. This approach aims to reduce bias and enhance the study's credibility.

**Comments about adverse effects:
· I understand after reading S1 file that some patients were hospitalized. Was there any evaluation of kidneys function after double preparation?**

**The author's answer:** Thank you for your valuable suggestions. Assessing renal function in patients after remedial bowel preparation is crucial for evaluating the safety of such measures. Based on your recommendation, we collected blood creatinine values from clinical records within one week before colonoscopy for all patients and after colonoscopy for some, including 33 patients in the hydrotherapy group (n=55) and 32 patients in the oral medication group (n=54). A total of 44 patients (22 in the hydrotherapy group and 22 in the oral medication group) did not have their blood creatinine levels checked shortly after colonoscopy. We used the endogenous creatinine clearance formula (Cockcroft-Gault) to estimate creatinine clearance before and after colonoscopy. Renal function was assessed using blood creatinine levels and endogenous creatinine clearance. Statistical analysis indicated no significant differences in blood creatinine levels or endogenous creatinine clearance between the two groups before colonoscopy, with baseline levels being comparable. This section was added to the lines 248-251 of the manuscript, and the data are shown in Table 1. After colonoscopy, differences in blood creatinine levels and endogenous creatinine clearance remained statistically insignificant for some patients in both groups. The difference in blood creatinine before and after colonoscopy and endogenous creatinine clearance within the hydrotherapy group were not significant, while creatinine clearance was higher post-colonoscopy in the oral medication group. Based on the current incomplete data analyses, we hypothesize that neither remedial bowel preparation method adversely affects patients' renal function. Given the incomplete data, this section was not explored in detail in the manuscript, but patient blood creatinine levels are included in the S1 file. Due to the small sample size and incomplete data, we plan to continue with a prospective study to obtain more comprehensive data.

| Variables | Hydrotherapy device enema group (n = 33) | | Oral medication group  (n = 33) | |
| --- | --- | --- | --- | --- |
|  | Before remedial bowel preparation | After remedial bowel preparation | Before remedial bowel preparation | After remedial bowel preparation |
| Serum creatinine | 72.94 ± 22.84 | 72.58 ± 24.24 | 77.13 ± 21.64 | 72.31 ± 19.34 |
| Endogenous Creatinine Clearance (Ccr) | 75.60 ± 20.25 | 76.12 ± 26.04 | 75.84 ± 22.56 | 81.94 ± 25.27* |

*Compared with this group before remedial bowel preparation，*P*﹤0.05

**· Colon hydrotherapy may cause rectal ulcers or other lesions, even if performed by a well-experienced nurse. Were there any lesions?**

**The author's answer:** Thank you for your valuable comments. In our retrospective analysis of clinical data from 54 patients who underwent bowel preparation using a hydrotherapy device enema, we observed that some patients experienced symptoms of anal itching and discomfort (n=8). However, there were no cases of rectal lesions or mucosal damage among the 54 patients. We attribute this to the strict training provided for the use of the hydrotherapy device. We ensured that the patient's anus was thoroughly lubricated before the procedure, and operators were instructed to perform the procedure gently, continuously monitor patient feedback, and avoid any forceful maneuvers. Additionally, the anal insertion part of the hydrotherapy device is designed to be rounded and smooth, which significantly reduces the risk of anorectal injury. We recognize the need to continue with prospective studies to increase the sample size and further validate these findings.

**Comments about the conclusions of the Authors:
· The first sentence (lines 393-394) is not well expressed. The increase of colonoscopies do not exactly increase the number of inadequate preparations. But it is true that having correct protocols and alternatives for colon preparation is a main step.**

**The author's answer:** We sincerely thank you for your careful review. Unclear statements can lead to misunderstandings, and we appreciate your feedback. We have made changes based on your suggestions and rephrased the statement as follows: "The proportion of patients with risk factors for inadequate bowel preparation is increasing in clinical practice."

**I have some suggestions about economical details to add in the Discussion Section, if it is possible, to give some more context to the article.**

**The author's answer:** Thank you very much for your valuable suggestions. Based on your input, we have analyzed and discussed the economic aspects in the discussion section of the manuscript. **· What is the difference between repeating the colonoscopy the same day or another day?**

**The author's answer:** For inadequate bowel preparation (insufficient to detect polyps larger than 5 mm), it is recommended to either terminate and reschedule the colonoscopy or attempt additional cleansing without canceling the examination. Patients must schedule their colonoscopy appointments in advance and complete blood tests and electrocardiograms to assess overall health prior to the procedure. Some patients may also need to discontinue anticoagulant and antiplatelet medications, as directed by the referring physician. Imagine a scenario where a patient, on the day of their colonoscopy, completes the procedure after opting for remedial bowel preparation due to inadequate initial preparation. This streamlined approach helps avoid re-booking or repeating pre-colonoscopy assessments and bowel preparation, thereby reducing repeat visits and associated costs, and ensuring timely, high-quality examinations. We have presented this information in the manuscript (lines 358-368).

**· I assume there is an extra agenda in the Endoscopy Unit to repeat a patient with poor / inadequate colon preparation. How is it managed? The goal is to avoid cancellation, but as care pressure is increasing and agendas are commonly tight, it is very difficult to me to understand how it is done.**

**The author's answer:** Thank you for your valuable comments. We apologize for any confusion caused by our lack of explanation and would like to provide clarification. The study population in this retrospective study consisted of inpatients scheduled for colonoscopy due to various indications. Our endoscopy center is currently integrated with the gastroenterology ward, which features a dedicated enema room equipped with a hydrotherapy machine. A nurse is tasked with operating the colonic hydrotherapy machine daily. When a colonic hydrotherapy enema is required, the attending physician coordinates with the nurse to ensure the procedure is conducted promptly. We recognize that, as this remedial bowel cleansing program is implemented in clinical practice, further investigation is needed into the coordination between the attending physician, endoscopy center, nursing staff, and patients. This will enhance the program's adaptability and facilitate its broader adoption. We have presented this information in the manuscript (lines 440-447, 452-455) **· What is the extra cost to have every day a trained nurse to perform colonic hydrotherapy, just in case it is needed?**

**The author's answer:** Thank you very much for your valuable comments. We apologize for any confusion caused by our lack of explanation and would like to provide clarification. Currently, our endoscopy center is integrated with the gastroenterology department, which includes a dedicated hydrotherapy enema room. A nurse is responsible for operating the colon hydrotherapy machine daily. This machine is used not only for bowel cleansing but also frequently for treating chronic constipation and administering medication retention enemas. When a patient requires a colon hydrotherapy enema, the attending physician coordinates with the nurse on duty to ensure the procedure is conducted promptly.We have presented this information in the manuscript (lines 440-447).

**Reviewer #3**

1. **I am not familiar with the term "electronic colonoscopy" is this just a standard colonoscopy or is this a variant?**

**The author's answer:** Thank you for your valuable feedback, and we apologize for any confusion caused by our unclear presentation. The term "electronic colonoscopy" refers to a standard colonoscopy. We have revised the manuscript to replace "electronic colonoscopy" with "colonoscopy" for clarity and simplicity.

2. **How did you select who received which intervention?**

**The author's answer:** We thank you for your helpful comments and apologize for not explaining this issue clearly in the article. In our daily clinical practice at the Gastroenterology Department and Endoscopy Centre, we perform an initial assessment of bowel cleanliness for patients undergoing colonoscopy on the day of the scheduled examination. This assessment is based on the patient's last bowel movement, and the results are recorded in the patient's medical record. The assessment was graded into four categories:

Grade 1: Very poor bowel preparation (brown fecal water with a large amount of waste material).

Grade 2: Relatively poor (dark yellow fecal water with a small amount of fecal waste material).

Grade 3: Good (yellowish clear watery stool).

Grade 4: Very good (colorless clear watery stool).

Grades 1 and 2 were considered inadequate bowel preparation. For patients assessed with Grades 1 and 2, we inform them of the potential adverse effects of insufficient bowel preparation and recommend remedial measures, while ultimately respecting the patient's decision. We offer patients optional solutions, including undergoing remedial bowel preparation, rescheduling a colonoscopy appointment, or proceeding with the colonoscopy attempt. A total of 121 patients with inadequate bowel preparation following routine administration (either single or divided oral intake) of polyethylene glycol electrolyte solution) for colonoscopy at Beijing Changping Hospital of Traditional Chinese Medicine between April 2019 and June 2022 were included in the study. Of these, 3 patients did not complete colonoscopy due to difficulties with scope access or intolerable abdominal pain, and 9 patients refused to participate in telephone follow-up. Consequently, 109 patients were included in the final analysis as detailed in lines 111-146 of the manuscript. We have also provided a flowchart (Figure 1) to illustrate the process.

3. **Were any of your satisfaction assessments validated tools?**

**The author's answer:** Thank you for your question. We apologize for not making this clear and complete in the manuscript. We have uploaded the telephone questionnaire as supplementary data (S2 File). The telephone questionnaire consisted of seven questions. The tool used for the satisfaction assessment part of the telephone follow-up was the Likert scale, which assessed patients' satisfaction with the bowel preparation process by rating the following three areas: (a) satisfaction with the time taken for the bowel preparation process; (b) tolerance of discomfort during the bowel preparation process; and (c) satisfaction with the service attitude of the medical staff during the bowel preparation process. Satisfaction was rated on a numerical scale from 0 (very dissatisfied) to 10 (very satisfied). We have presented this information in the manuscript (lines 218-225).

4. **Can you expand on how many patients were sent for remedial therapy solely based on the passage of stool just prior to their scheduled procedure vs how many had the scope placed and the BBPS evaluated? For those where the scope was placed and the BBPS was evaluated for at least 1 segment was there a difference in the change in prep quality adjustment between the 2 interventions?**

**The author's answer:** Thank you for your valuable comments, and we apologize for not stating this clearly in the manuscript. In this retrospective study, one of the proposed inclusion criteria for patients was inadequate bowel preparation, defined as the absence of the required characteristics—clear watery stool or clear yellowish liquid—after administration of the original bowel preparation protocol. Alternatively, bowel preparation was also deemed inadequate if assessed during colonoscopy with a BBPS of less than 6 or a score of less than 2 in any of the three segments of the colon. However, during data collection, we discovered that the final 109 patients included in the study were judged to have failed bowel preparation based solely on their final bowel movement after routine preparation. There was no clinical data record indicating these patients underwent colonoscopy due to a failure in the BBPS assessment after routine bowel preparation. In lines 125-135 of the manuscript, we have addressed and clarified this issue. Thank you again. We think this is an excellent suggestion.

5. **How do you define preprocedureal constipation for the purposes of this study?**

**The author's answer:** We take the issue you have raised very seriously. The diagnosis of preprocedureal constipation primarily relies on symptoms. Constipation can be diagnosed when there is difficulty and effort in defecation, fewer than three bowel movements per week, and dry, small stools. Chronic constipation is defined as symptoms persisting for six months or longer. The diagnosis of chronic functional constipation is currently based on the Rome IV diagnostic criteria, which include the following: (1) Two or more of the following symptoms must be present in at least 25% of defecations: laborious defecation, dry pellet-like or hard stools, a sensation of incomplete evacuation, a feeling of anorectal obstruction and/or blockage, and the need for manual assistance during defecation, with spontaneous defecation occurring less than three times per week. (2) Loose stools are rarely present without the use of laxatives. (3) The condition does not meet the diagnostic criteria for irritable bowel syndrome.

6. **What is the definition for this study of "success rate" of the bowel preparation?**

**The author's answer:** Thank you for your question. We sincerely apologize for not defining "success rate" within the manuscript. In this study, the Boston Bowel Preparation Scale (BBPS) was utilized to evaluate the quality of bowel preparation in patients undergoing salvage bowel preparation. The BBPS is a standardized 9-point scale for assessing the colon, which is divided into three segments: right colon, transverse colon, and left colon. Each segment is rated from 0 to 3 based on the degree of cleanliness. The total score, which ranges from 0 to 9, indicates the level of bowel preparation: ≤5 points indicates poor preparation, 6–7 points indicates good preparation, and ≥8 points indicates very good preparation. A BBPS score of ≥6 was considered satisfactory. The success rate was defined as the proportion of patients in each group achieving a BBPS score of ≥6. The definition of success rate has been added to the manuscript at lines 204-205.

7. **What is the difference in cost between these two measures?**

**The author's answer:** Thank you very much for your valuable comments. We have calculated the cost of the two bowel cleansing protocols. In our healthcare system, the cost of administering 2 liters of Compound Polyethylene Glycol Electrolyte Powder orally is RMB 69.3, whereas a hydrotherapy enema costs RMB 56. Thus, hydrotherapy enema for bowel preparation is less expensive for patients compared to the oral administration of Compound Polyethylene Glycol Electrolyte Powder. We have presented this information in the discussion section of the manuscript(lines 454-460) and hope to conduct further studies to compare cost-effectiveness of these two methods.

Thank you very much for your attention and time. Look forward to hearing from you.

Yours sincerely,

Dongxuan Zhang

25 December 2024
