# Supplementary material for: Remedial colon hydrotherapy device enema as a salvage strategy for inadequate bowel preparation for colonoscopy: A retrospective cohort study
Source: PLoS One. 2025 Mar 19;20(3):e0319493. doi: 10.1371/journal.pone.0319493 (PMC11922272; doi:10.1371/journal.pone.0319493)
Supplement: Attachment — Submitted filename: A_rebuttal_letter_auresp_2.docx [file pone.0319493.s008.docx]

Dear Editors and Reviewers,

We would like to express our sincere gratitude to the editors and reviewers for their thorough examination of our manuscript and for providing valuable and constructive feedback. We deeply appreciate the time and effort invested in reviewing our work, and we are confident their insights will substantially enhance the quality of our study.

Our article, titled "Remedial Colon Hydrotherapy Device Enema as a Salvage Strategy for Inadequate Bowel Preparation for Colonoscopy: A Retrospective Cohort Study" (Manuscript No: PONE-D-24-32926), explores strategies to improve bowel preparation—a crucial component of effective diagnostic procedures for colonoscopy. We are pleased that the reviewers have acknowledged the significance and novelty of our research. Their suggestions have been instrumental in refining and clarifying our findings. We have carefully considered each of their comments and made the necessary revisions to address the issues raised. For your convenience, all changes in the revised manuscript are highlighted in red text. We have rechecked the reference list of the manuscript to ensure its completeness and accuracy. The 13th and 22nd references in the article are in Chinese, and we have labeled them in the reference list according to ICMJE recommendations（line 520 and line 551）.

Below this letter, you will find point-by-point responses to the reviewers' comments.

We believe these revisions have significantly strengthened our manuscript, and we appreciate the opportunity to address these important concerns.

**Reviewer #1: (No Response)**

**Reviewer #2: Thank for allowing to review this manuscript. The Authors have gone through all points expressed by the Reviewers very carefully.
I have some minor comments to give:
· Lines 82-83: these medications can induce secondary effects in the digestive tract, such as nausea, vomiting and (add) “alterations in intestinal transit”.**

**The author's answer**: Thank you for your valuable comments. Your revision has made this statement more comprehensive and precise. We have incorporated this change into the manuscript (lines 83-84). **· Lines 118-119: I am confused with the sentence “The use of tryciclic antidepressants or anesthesia also increases this risk”. Is “anesthesia” referring to analgesia / pain killers?**

**The author's answer**: We thank you for your helpful comments and apologize for not explaining this issue clearly in the article. In the manuscript, “anesthesia” is referring to narcotic analgesics, such as opioids. For greater clarity and to avoid misunderstandings, we have revised this line in the manuscript to read: "The use of tricyclic antidepressants or narcotic analgesics, such as opioids, also increases the associated risks." (lines 118-119)

**· Line 182: I would separate in different paragraphs these sentences: one paragraph for hydrotherapy, and one for oral medication.**

**The author's answer**: Thank you for your valuable advice. This revision enhances the article's structure. We have reorganized this section of the manuscript into two paragraphs: one focused on hydrotherapy and the other on oral medication. (lines 178-195) **· Line 446: I would separate in different paragraphs these sentences: one paragraph for the hospital organization, one for comments about the study.**

**The author's answer**: Thanks again for your valuable comment. We have reorganized this section of the manuscript into two paragraphs: one focused on the hospital organization and the other on comments about the study. (lines 446-467) **· In my opinion, the conclusion given is too aggressive. The results of this retrospective study lead to think that colon hydrotherapy is a good and safe remedial therapy for patients with inadequate bowel preparation, but there is no strength in data to say that “it is recommended as the optimal remedial strategy”. I would be more cautious with this sentence.**

**The author's answer**: Thank you for your valuable comments. We fully understand your concerns. Indeed, in this retrospective study, the conclusions were initially expressed too assertively. Based on the current study results, we recognize the need to conduct further prospective studies to validate our conclusions. Following your comments, we have revised the abstract and conclusion sections of the manuscript, which now state: "It is recommended as an effective and safe remedial therapy for patients with inadequate bowel preparation prior to colonoscopy." (lines 64-65) and "Consequently, it is an effective and safe remedial therapy for patients with inadequate bowel preparation prior to colonoscopy." (lines 477-478).

**Reviewer #3: Thank you for the opportunity to review the revised manuscript, "Remedial colon hydrotherapy device enema as a salvage strategy for inadequate bowel preparation for colonoscopy: A retrospective cohort study." Please find my comments below:**

**1. You list as exclusion criteria "Patients who used two or more remedial options in combination." Could you be more clear here. I agree that if they used both techniques without an evaluation between each technique it would be appropriate to exclude. However, if they used one method and had an inadequate prep necessitating use of the other technique, then they should be included as a treatment failure.**

**The author's answer**: Thank you for your valuable comments, and we apologize for not clearly presenting this in the manuscript. In this retrospective clinical study, we included "patients with a combination of two or more remedies" as an exclusion criterion. In clinical practice, the specifics of combining two or more therapeutic regimens include the following scenarios:

1. After routine bowel preparation, if a patient is initially assessed as having inadequate bowel preparation, the treating clinician may first administer a low dose of polyethylene glycol (PEG) electrolyte powder orally. If the patient is unable to continue oral intake due to severe nausea, vomiting, or other adverse reactions, or if there is no bowel movement post-intake, the clinician may communicate with the patient to proceed with either a plain cleansing enema or a colon hydrotherapy device enema to continue the bowel preparation.
2. Patients evaluated as having poor bowel preparation via fecal assessment, who did not respond to a plain cleansing enema, might receive a colon hydrotherapy device enema.

These patients undergo colonoscopy following a complex combination of bowel preparation methods, with cleanliness assessed using the BBPS scale. We determined that it was challenging to ascertain whether the bowel cleanliness was due to a specific bowel preparation regimen or the combined effect of different regimens. Therefore, we excluded patients who used combinations of two or more bowel cleansing regimens in this retrospective clinical trial. This explanation is provided in lines 167-170 of the manuscript.

Thank you again for your suggestion, which has highlighted the need to further investigate and validate the scope of colon hydrotherapy device enema as a remedial bowel preparation method. This includes not only its use for inadequate bowel preparation following conventional regimens but also exploring its value after other remedial preparation regimens.

Once again, we thank the reviewers for their support and for their role in helping us to improve our manuscript. We look forward to the opportunity to further discuss our work with you.

Yours sincerely,

Dongxuan Zhang

17 January 2025
